# The So-Called Crisis in the University of London

**Published:** 1907-05-11

**Authors:** 


### The Hospital

A Journal of

The Medical Sciences and Hospital Administration.

New Series. No. 6, Vol. I. [No. 107[illegible], Vol. XLII.] SATURDAY, MAY 11, 1907.

### THE SO-CALLED CRISIS IN THE UNIVERSITY OF LONDON.

The interest and excitement aroused by the recent Senatorial Election in the Faculty of Medicine have not diminished, and in the voluminous correspondence which has appeared in the fly-leaves and circulars with which University voters are being bombarded, many conflicting policies are revealed. The supporters of the "concentration policy," surprised and hurt at the base ingratitude of the electors of the Faculty of Medicine, are trying to prove by means of historical accounts of the previous action of the Senate and of the Faculty of Medicine, particularly, that, the University has been stultified and degraded by a flippant and irresponsible reversal of policy. Indeed the electorate of the Faculty of Medicine are accused of having encouraged the prosecution of a certain policy for years, of having collected a large sum of money for an Institute of the medical sciences, and of then, at the eleventh hour, deciding that a Central Medical Institute no longer meets with its approval. These statements will not bear the test of investigation, for, as we stated in The Hospital of March 23, the policy of the University, upon which the last election in the Faculty of Medicine was fought, was the erection of a third centre at South Kensington, for the teaching of the preliminary and intermediate subjects, University and King's Colleges being also available for that purpose.

It is true the majority of the Medical Schools expressed an opinion in favour of the concentration of the early studies, and would probably repeat their adherence to this scheme, if they were consulted again to-morrow; but it is a very different thing to have expressed adherence or approval of such a scheme in the abstract, and to be bound by that academic opinion to any detailed and particular scheme which the University, or a party in the University, may have evolved without the assent of the Medical Schools.

The opposition aroused by the three-centre scheme would seem at first sight to be out of all proportion to the facts, but it is the more strenuous because it is felt that, the party who are advocating this project have never taken either the electors or the public properly into their confidence. In the historical précis which has recently appeared in a contemporary, there is no mention of internal examinations; yet it is well known and has never been denied, that, the institution of internal examinations is an integral part of the three-centre scheme, and it is the institution of internal examinations to which many members of the University take exception.

It is felt that, human nature being what it is, internal examinations are bound to end in the degradation of the standard of the Degrees at present granted by the University, for it is a well known fact that examiners are always liable to mark the candidates whom they have personally taught more highly than strangers, as their own pupils naturally answer questions in a manner attractive to their teachers. So competition between one or more centres, each with its own internal examinations, might probably be the starting point of a decline from the present high standard of fairness in the Examinations of the University of London. Again, it is obvious to every School which has no endowments, which will not be favoured with the institution of internal examinations, that the centres supported out of the University funds and favoured by the privilege of internal examinations, may sooner or later freeze out all the other Medical Schools, whether efficient or not. It is therefore hardly likely that, the University can expect the cordial support of the Medical Schools, who see that such a scheme as this may tend to their eventual disadvantage.

Because the Faculty of Medicine has disapproved of the University's policy, it is presumed that this Faculty also disapproves of the policy of the University generally, and violent and undeserved attacks are being made upon the Matriculation Examination and other Examinations for the Medical Degree. The attacks made upon the Matriculation Examination are, however, quite unjustified. Besides, very wise and liberal arrangements have been made with the older Universities whereby certain examinations taken there are regarded as satisfactory substitutes for the Matriculation of the University of London. In fact, the whole policy of the University with reference to Matriculation has been wise and liberal, and has in no case merited attack. Further, the University can be trusted, with the greatest confidence, to keep up the standard of the Final Examinations in the future.

It is apparent that so many lines of cleavage have been formed by the recent unfortunate developments of the University's policy with reference to the teaching of the preliminary and intermediate subjects that the time has come for the formation of a new party among the Graduates, a party which recognises the danger of internal examinations and of any scheme of concentration which has no greater magnitude than the building of an Institute, not larger than some of the more competent Medical Schools; that will prevent the Incorporated Colleges of the University having too great an accession of privilege without proper supervision, that will support the University's present liberal policy with reference to Matriculation and the Examinations for Degrees; but will at the same time urge the necessity of inspecting all the Medical Schools of the University, in order that the Senate may have reliable information as to their efficiency.

There are many well known and reputable Members of the university, who are not in favour of the policy of concentration at three centres, but who are yet opposed to the tactics of the reactionary party in the Senate, and who would be only too willing to join any body of Graduates which desired the continuance of the great traditions of the University of London, without sacrificing the efficient Medical Schools now recognised by the University. The election of Sir Thomas Barlow would seem to indicate that, the trend of opinion is in favour of the policy of the new party, as we have indicated it.

